# Supplementary material for: Dyes Adsorption Behavior of Fe3O4 Nanoparticles Functionalized Polyoxometalate Hybrid
Source: Molecules. 2019 Aug 28;24(17):3128. doi: 10.3390/molecules24173128 (PMC6749234; doi:10.3390/molecules24173128)
Supplement: Supplementary file 1 [file molecules-24-03128-s001.pdf]

Dyes Adsorption Behavior of Fe_3O_4 Nanoparticles Functionalized Polyoxometalate Hybrid

Jie Li, Chen Si, Haiyan Zhao, Qingxi Meng, Bowen Chang, Mingxue Li* and Hongling Liu*

Henan Key Laboratory of Polyoxometalates Chemistry, Institute of Molecular and Crystal Engineering, College of Chemistry and Chemical Engineering, Henan University, Kaifeng 475004, Henan, China

* Correspondence: limingxue@henu.edu.cn (M.X.L.), hlliu@henu.edu.cn (H.L.L.)

Content:

Section 1: Crystallographic data for 1.
Section 2: The related equations.
Section 3: Thermogravimetric analyses of 1, Fe_3O_4@1 and Fe_3O_4.
Section 4: Adsorption activity comparison of 1, Fe_3O_4@1 and Fe_3O_4.

Section 1: Crystallographic data for 1

	**1**
Formula	$C_{28}H_{42}Mo_5N_{20}Ni_2O_{25}P_2S_4$
F_w	1846.05
Crystal system	Monoclinic
space group	*C2/c*
a/[Å]	30.575(7)
b/[Å]	13.276(3)
c/[Å]	17.009(4)
β[°]	113.837(5)
Z	4
Volume/[$Å^3$]	6315(3)
Calculated bulk density/[g cm^{-3}]	1.940
Absorption coefficient/[mm^{-1}]	1.814
F(000)	3632
Limiting index	−20≤h≤36, −15≤k≤15, −20≤l≤19
Theta range of data collection	1.70 to 25.00°
Limiting index	−20≤h≤36, −15≤k≤15, −20≤l≤19
Reflections collected/unique	15775/5569 [R(int) = 0.0686]
Data/restraints/parameters	5569/0/385
Goodness-of-fit on F^2	1.054
Final *R* indices [I > 2σ(I)]	R_1 = 0.1755, wR_2 = 0.3979
R indices (all data)	R_1 = 0.2297, wR_2 = 0.4257
Largest diff. peak and hole/[e ·Å^{-3}]	4.763, –4.493

Section 2. The related equations.

$$\text{Removal efficiency (\%)} = \frac{A_0 - A_t}{A_0} \qquad (1)$$

$$q_e = \frac{(C_0 - C_e)\times V}{W} \qquad (2)$$

where C_0 and C_t are the dyes concentration (mg/L) at initiation and the time t. A_0 and A_t are the dye absorbance at initiation and the time t. *w* and *V* is the dosage of the adsorbent (mg) and the amount of dye (mL), respectively.

$$\log(q_e - q_t) = \log q_e - k_1 t \qquad (3)$$

$$\frac{t}{q_t} = \frac{1}{k_2 (q_e)^2} + \frac{t}{q_e} \qquad (4)$$

where q_t is the adsorption capacity of MB at time t. k_1 is the rate constant of pseudo-first-order, k_2 is the rate constant of pseudo-second-order.

$$\frac{C_e}{q_e} = \frac{C_e}{q_m} + \frac{1}{K_L q_m} \qquad (5)$$

$$lgq_e = lgK_f + \left(\frac{1}{n}\right) lgC_e \qquad (6)$$

where q_m and C_e are the maximum adsorption capacity and equilibrium concentration of MB in the solution, respectively. K_L is the Langmuir constant. K_F is the Freundlich constant. n is the adsorption strength.

Section 3: Thermogravimetric analyses of 1, Fe_3O_4@1 and Fe_3O_4.

Thermogravimetric analyses of **1**, Fe_3O_4@**1** and Fe_3O_4 were performed under a nitrogen flow (Figure. S1). It turns out that the weight ratio of **1** in Fe_3O_4@**1** is 89.57%. It should be noted that the loss of Fe_3O_4 might be attributed to its surfactant attached during the synthesis process.

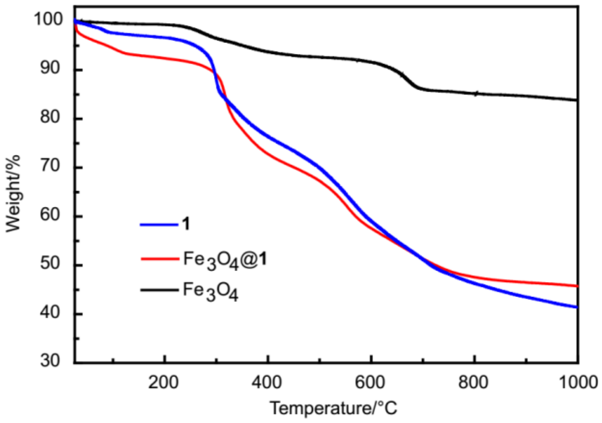

Figure S1. Thermogravimetric analyses of **1**, Fe_3O_4@**1** and Fe_3O_4.

Section 4: Adsorption activity comparison of 1, Fe_3O_4@1 and Fe_3O_4.

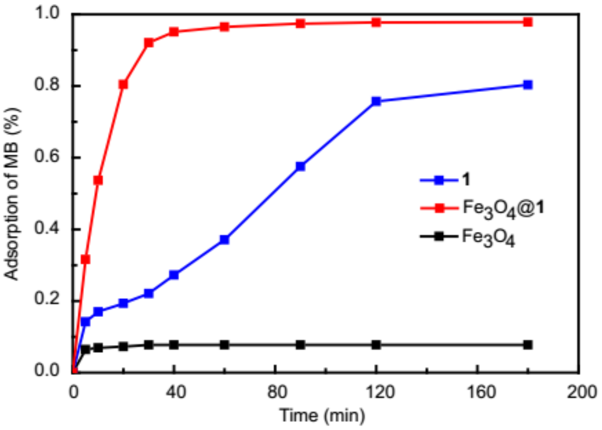

Figure S2. Adsorption activity comparison of **1**, Fe_3O_4@**1** and Fe_3O_4. (MB: 15 mg/L, 10 mL; room temperature)
